# Synergistic Al^3+^ Doping and Multilayer ZnS Shell for Enhanced Photoluminescence and Long-Term Stability of Water-Soluble ZnSe Quantum Dots

**DOI:** 10.3390/nano16171057

**Published:** 2026-08-25

**Authors:** Xiaoyan Li, Duo Chen, Chaojin Xuan, Boxu Yang, Qingyu Hai

**Affiliations:** Smart Materials Laboratory, Department of Applied Physics, Northwestern Polytechnical University, Xi’an 710129, China

**Keywords:** Al-doped ZnSe QDs, multilayer ZnS shell, stepwise shell growth, photoluminescence, long-term stability, water-soluble quantum dots

## Abstract

Water-soluble ZnSe quantum dots (QDs) typically suffer from limited photoluminescence (PL) efficiency and poor long-term stability due to intrinsic defects and surface traps. Here, we report a strategy combining Al^3+^ doping with stepwise multilayer ZnS shell growth. The stepwise precursor injection ensures uniform shell formation, while Al^3+^ incorporation suppresses defect-related recombination within the ZnSe core. The resulting ZnSe:Al/ZnS/ZnS/ZnS QDs exhibit a PL quantum yield of 23.24% and an average lifetime of 129.28 ns. They retain 91% of their initial PL intensity after 200 days under ambient conditions. Mechanistic analysis indicates that defect regulation and multilayer shell passivation synergistically promote band-edge radiative recombination over defect-assisted pathways. This work provides a strategy for simultaneously regulating defect states and surface stability in water-soluble semiconductor QDs.

## 1. Introduction

ZnSe quantum dots are promising environmentally benign luminescent nanomaterials owing to their low toxicity, tunable optical properties, and broad potential in bioimaging, biosensing, and optoelectronic applications [1,2,3,4]. Although ZnSe-based nanocrystals synthesized in nonpolar organic solvents have achieved near-unity PLQYs through advanced shell engineering strategies [5], water-soluble ZnSe QDs still face challenges in achieving high emission efficiency and long-term stability due to aqueous synthesis-induced defects and environmental sensitivity [6,7,8,9]. Overcoming these limitations is essential for the practical application of water-soluble ZnSe QDs, especially in long-term optical devices and bioapplications.

Doping engineering offers an effective approach to improve the optical performance of ZnSe QDs by regulating their local electronic environment and suppressing nonradiative energy losses [10,11,12,13]. Among various dopants, Al^3+^ has attracted particular interest because its small ionic radius and aliovalent nature allow it to substitute Zn^2+^ sites and modify the electronic structure of ZnSe. Previous studies on Al-doped ZnSe films have demonstrated that Al incorporation can effectively modulate the electrical and optical properties of ZnSe materials [14,15,16]. Nevertheless, these studies have primarily focused on thin-film systems fabricated by sophisticated techniques such as molecular beam epitaxy and laser-assisted deposition. Translating such defect-regulation strategies to water-soluble ZnSe QDs remains challenging because aqueous synthesis introduces abundant surface and interface defects.

Beyond doping within the ZnSe core, surface passivation through the ZnS shell has emerged as another effective strategy to enhance photoluminescence by suppressing surface-related nonradiative recombination and improving carrier confinement [17,18,19,20]. Nevertheless, recent studies have revealed that the effectiveness of shell passivation is critically governed by shell architecture and growth quality [21,22,23]. Nonuniform shell growth and incomplete surface coverage may leave residual interfacial defects, which continue to act as nonradiative recombination centers and thereby limit further improvements in luminescence efficiency and stability [24,25]. Therefore, developing controllable shell-growth strategies remains essential for maximizing the optical performance of water-soluble ZnSe quantum dots.

Herein, we propose a strategy that integrates Al^3+^ doping with ZnS shell engineering to simultaneously improve the photoluminescence performance and long-term stability of water-soluble ZnSe quantum dots. Al^3+^ doping is incorporated into the ZnSe core to modulate defect-related recombination, while a stepwise precursor injection method enables engineered multilayer ZnS shell formation for effective surface passivation. This work provides a versatile strategy for the rational design of water-soluble quantum dots with enhanced photoluminescence performance and stability.

## 2. Experimental Section

### 2.1. Reagents

All reagents employed in the experiments are listed in Table 1 and were of analytical-grade purity.

### 2.2. Preparation

(1) Pre-experiment preparation

The ZnSe:Al core precursor solution was prepared by mixing 40 mL of 0.1 mol L^−1^ Zn(NO_3_)_2_·6H_2_O aqueous solution with Al(NO_3_)_3_·6H_2_O (0.028 mmol) and Mercaptopropionic acid (MPA, 4.8 mmol) under continuous stirring. The molar ratio of Zn^2+^, Al^3+^, and MPA was controlled at 2:0.014:2.4.

The Se precursor was prepared by reacting selenium powder with NaBH_4_. Briefly, NaBH_4_ (27 mmol) was dissolved in 18 mL deionized water, followed by the addition of selenium powder (9 mmol). After complete conversion of selenium powder, a NaHSe solution (0.5 mol L^−1^) was obtained and used as the Se precursor.

A ZnS precursor solution was obtained by dissolving Zn(Ac)_2_·2H_2_O (8 mmol), thiourea (8 mmol) and MPA (12 mmol) in deionized water, with the molar ratio of Zn^2+^, S^2−^, and MPA controlled at 1:1:1.5.

(2) ZnSe:Al/ZnS/ZnS/ZnS QDs preparation

**Figure 1:**
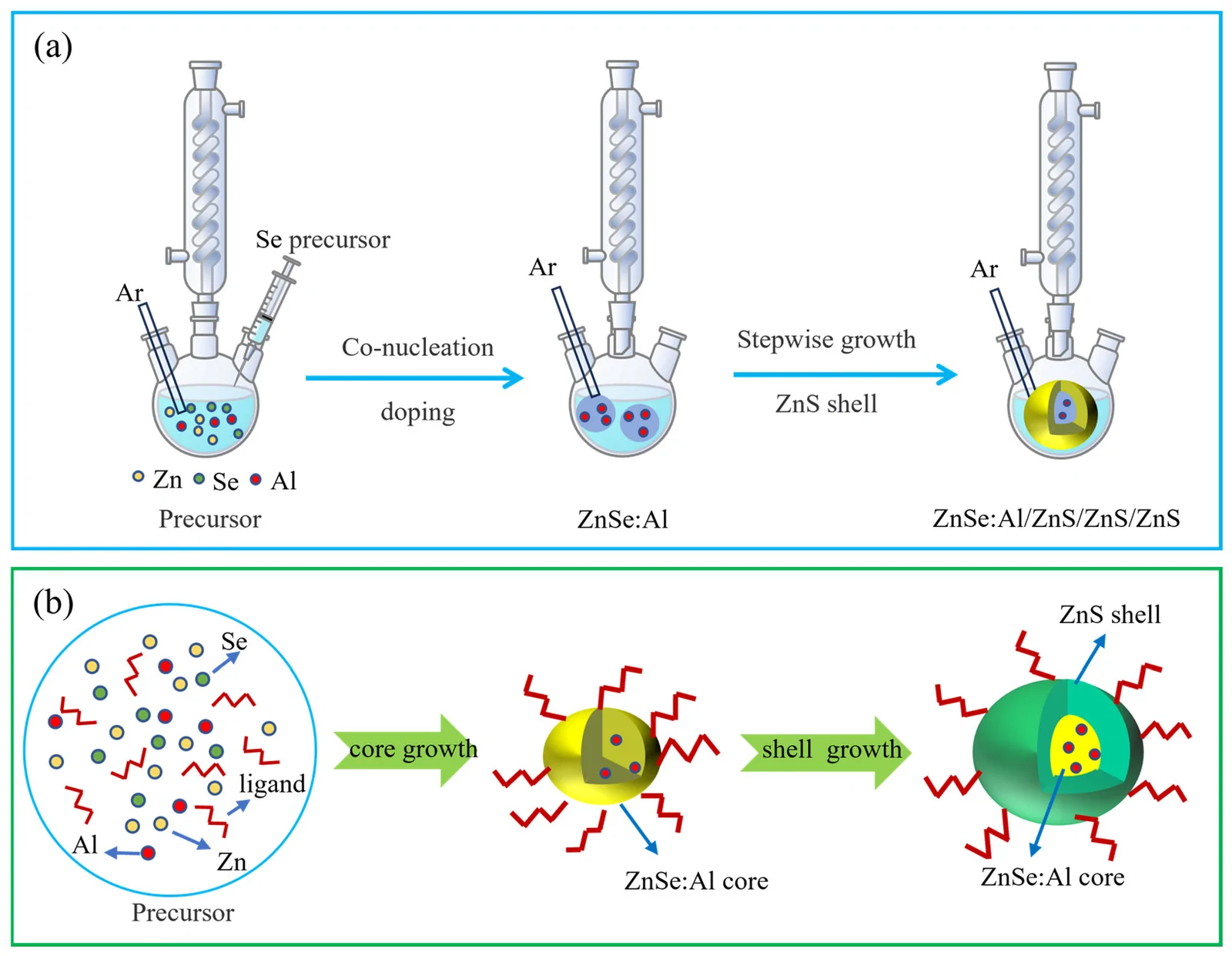
Stepwise synthesis strategy and growth mechanism of ZnSe:Al/ZnS/ZnS/ZnS QDs. (**a**) Synthesis flowchart of ZnSe:Al/ZnS/ZnS/ZnS QDs. (**b**) Schematic diagram of the corresponding growth mechanism.

Generating ZnSe:Al/ZnS/ZnS/ZnS QDs involves a multi-step process, as illustrated in Figure 1. Initially, the cationic precursor solution with a pH of 10.30 was obtained by adding a 10%NaOH solution. After stirring under an Ar atmosphere for 30 min, 4 mL of NaHSe precursor solution was rapidly injected into the precursor solution at room temperature. The reaction mixture was then heated to 100 °C and vigorously stirred for 2.5 h to obtain ZnSe:Al QDs. To prepare ZnSe:Al/ZnS/ZnS/ZnS QDs, the ZnSe:Al QD solution was first cooled to room temperature, followed by the sequential addition of equal amounts of ZnS precursor solution. After each precursor injection, the pH of the reaction mixture was adjusted to 10.00 using 10 wt% NaOH solution, and the mixture was maintained at 90 °C under an Ar atmosphere for 1.5 h to allow for the growth of each ZnS shell layer. This sequential shell-growth process was repeated three times to obtain the final ZnSe:Al/ZnS/ZnS/ZnS QDs.

The obtained QDs were precipitated with ethanol, collected by centrifugation, washed three times with ethanol and deionized water, and vacuum-dried to obtain QD powders. All syntheses were independently repeated at least three times, and similar optical properties were obtained.

### 2.3. Characterizations

The transmission electron microscope (TEM) images and size of the prepared QDs were recorded by FEI Talos F200X (Thermo Fisher Scientific, Waltham, MA, USA)and Malvern Zetasizer Nano ZS90 (Malvern Instruments Ltd., Malvern, UK). The crystal structure, chemical structure and elemental composition of the prepared QDs were characterized by XRD-7000 diffractometer (Shimadzu Corporation, Kyoto, Japan), FT/IR-400 plus VIR-9000 Fourier spectrometer (JASCO Corporation, Tokyo, Japan), and Axis Supra X-ray photoelectron spectrometer (Kratos Analytical Ltd., Manchester, UK), respectively. The optical properties of the QDs are evaluated by ultraviolet-visible spectrophotometer (UV-3600, Shimadzu Corporation, Kyoto, Japan), and fluorescence spectrophotometer (F-7000, Hitachi High-Technologies Corporation, Tokyo, Japan), Edinburgh FLS 980 time-resolved fluorescence spectrometer (Edinburgh Instruments Ltd., Livingston, UK).

## 3. Results and Discussion

**Figure 2:**
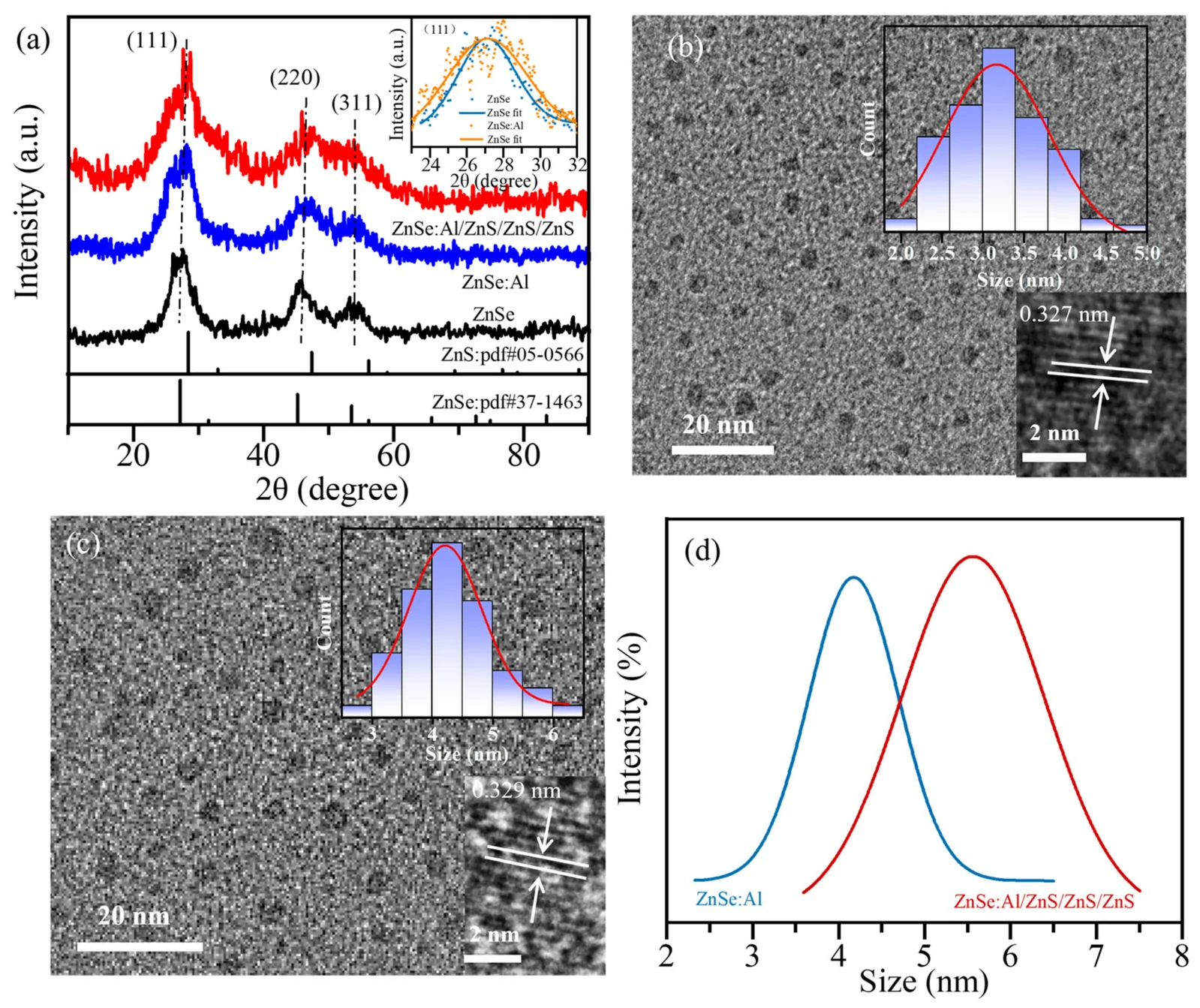
Structural characterization of the synthesized QDs. (**a**) XRD patterns of ZnSe, ZnSe:Al, and ZnSe:Al/ZnS/ZnS/ZnS QDs. The inset presents the pseudo-Voigt fitting results of the (111) diffraction peaks of ZnSe and ZnSe:Al QDs for quantitative structural analysis. (**b**) TEM image of ZnSe:Al QDs with the corresponding particle size distribution histogram and HRTEM image shown as insets. (**c**) TEM image of ZnSe:Al/ZnS/ZnS/ZnS QDs with the corresponding particle size distribution histogram and HRTEM image shown as insets. (**d**) Hydrodynamic size distributions of ZnSe:Al and ZnSe:Al/ZnS/ZnS/ZnS QDs obtained from DLS measurements.

Figure 2a displays the X-ray diffraction (XRD) patterns of the as-synthesized QDs. All diffraction peaks can be indexed to the cubic zinc blende ZnSe phase (PDF#37-1463), confirming that neither Al doping nor ZnS shell growth alters the intrinsic crystal structure of ZnSe. Compared with ZnSe QDs, the diffraction peaks of ZnSe:Al QDs exhibit noticeable broadening after Al doping. To quantitatively evaluate these changes, pseudo-Voigt peak fitting was performed for the XRD patterns. The results reveal increased FWHM values, reduced apparent crystallite sizes, and a slight decrease in the lattice parameter after Al doping (Table 2). These structural changes suggest that Al^3+^ incorporation modifies the ZnSe lattice structure. Considering the smaller ionic radius of Al^3+^ compared with Zn^2+^, the lattice contraction is consistent with the possible substitution of Zn^2+^ sites by Al^3+^ [26,27,28]. Moreover, the slight shift in the diffraction peaks toward larger angles after ZnS shell growth suggests the formation of a ZnSe/ZnS core–shell structure rather than a simple physical mixture [18].

To evaluate the morphology and dispersion of the prepared QDs, TEM and dynamic light scattering (DLS) measurements were performed (Figure 2). TEM images reveal that both ZnSe:Al and ZnSe:Al/ZnS/ZnS/ZnS QDs exhibit spherical morphologies. The measured interplanar spacings, determined from multiple lattice fringe measurements in the HRTEM images, are 0.327 ± 0.002 nm for ZnSe:Al QDs and 0.329 ± 0.003 nm for ZnSe:Al/ZnS/ZnS/ZnS QDs, respectively. These values are consistent with the (111) planes of cubic ZnSe (Figure 2b,c). Analysis of the particle size distributions shows that the average diameter increases from 3.16 nm for ZnSe:Al cores to 4.21 nm for ZnSe:Al/ZnS/ZnS/ZnS QDs. This increase is consistent with the calculated thickness of three ZnS monolayers (~0.31 nm per layer, derived from the lattice constant α = 0.54 nm using Equation (1)), confirming the formation of the core/shell structure [29,30,31]. Complementary dynamic light scattering measurements further confirm the size evolution of the QDs, yielding hydrodynamic sizes of 4.17 nm for ZnSe:Al and 5.59 nm for ZnSe:Al/ZnS/ZnS/ZnS (Figure 2d). The slightly larger values compared with the TEM results arise from the hydrated ligand shell and the electrical double layer surrounding the nanoparticles in aqueous media [12].

(1) $$d=\sqrt{3}\alpha /3$$

d: average thickness of monolayer ZnS.

*α*: lattice constant of ZnS (0.54 nm).

**Figure 3:**
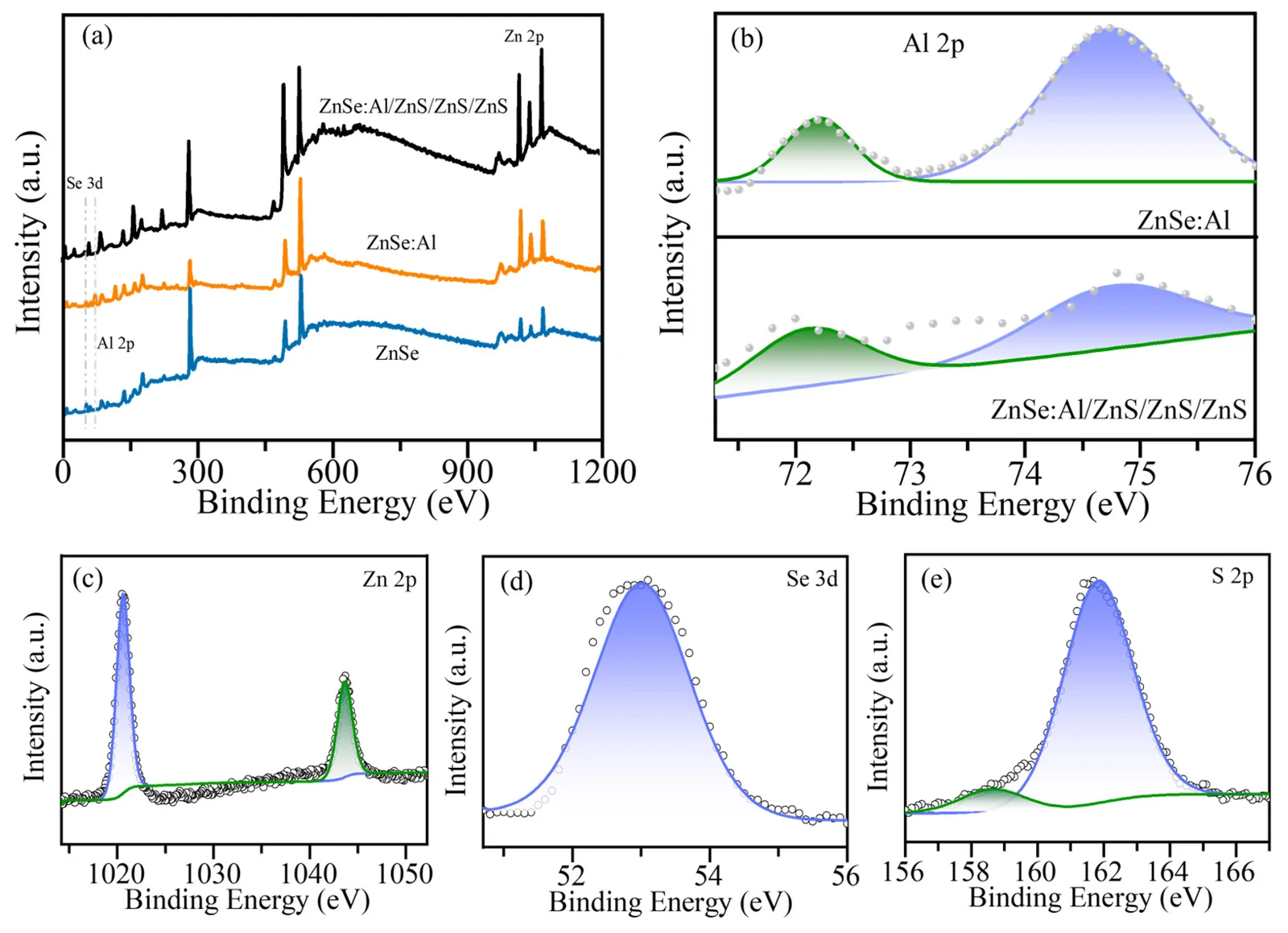
XPS characterization of ZnSe-based QDs. (**a**) Survey spectra of ZnSe, ZnSe:Al, and ZnSe:Al/ZnS/ZnS/ZnS QDs. High-resolution XPS spectra of (**b**) Al 2p, (**c**) Zn 2p, (**d**) Se 3d, and (**e**) S 2p for ZnSe:Al/ZnS/ZnS/ZnS QDs.

The XPS spectra of ZnSe:Al and ZnSe:Al/ZnS/ZnS/ZnS QDs (Figure 3) provide detailed information on the chemical states and the presence of the ZnS shell. Compared with pristine ZnSe QDs, both samples exhibit characteristic Al 2p peaks at 74.8 eV and 72.1 eV, confirming the successful introduction of Al species during synthesis [32]. Combined with the XRD results, these findings suggest that Al^3+^ is involved in regulating the ZnSe lattice rather than being exclusively associated with surface species. Meanwhile, the Zn 2p and Se 3d signals remain nearly unchanged in both samples, suggesting that the ZnSe crystal framework is well-preserved after Al doping and sequential ZnS shell growth. In the ZnSe:Al/ZnS/ZnS/ZnS QDs, the Al 2p signal is significantly attenuated, suggesting that the outer ZnS shell effectively encapsulates the ZnSe:Al core [33]. Moreover, ZnSe:Al/ZnS/ZnS/ZnS QDs display a distinct S 2p at ~161.8 eV, corresponding to S^2−^ species in ZnS, confirming the formation of the ZnS shell [34,35,36]. ICP analysis further quantified the Al content, revealing Al concentrations of 0.43% and 0.18% for ZnSe:Al and ZnSe:Al/ZnS/ZnS/ZnS QDs, respectively, confirming the presence of Al species in both core and core/shell structures.

**Figure 4:**
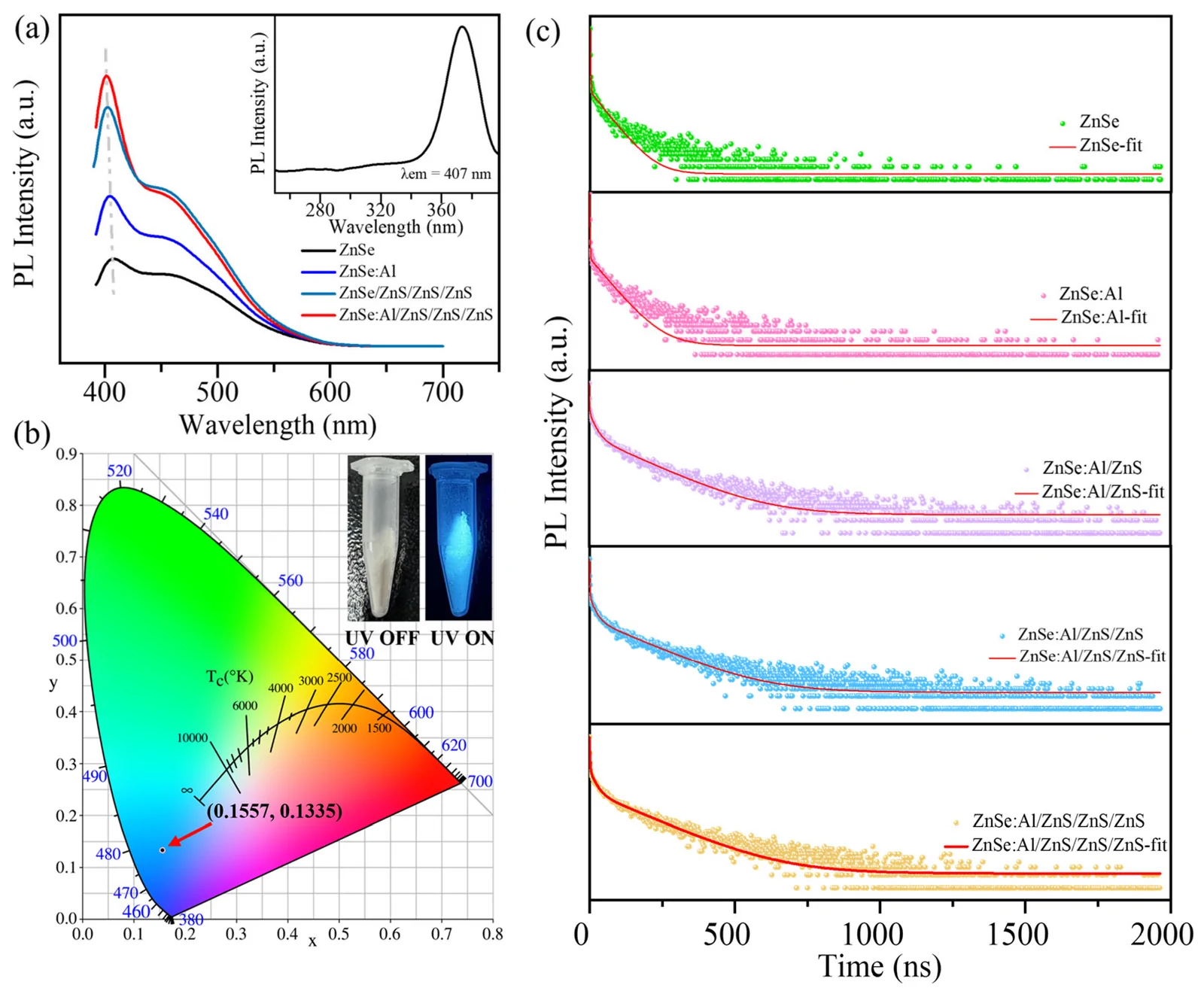
Optical characterization of the synthesized ZnSe-based QDs. (**a**) PL spectra of ZnSe, ZnSe:Al, ZnSe/ZnS/ZnS/ZnS and ZnSe:Al/ZnS/ZnS/ZnS QDs (λ_ex_ = 375 nm). The inset shows the PLE spectrum of the ZnSe:Al/ZnS/ZnS/ZnS QDs (λ_em_ = 407 nm). (**b**) CIE chromaticity diagram of the ZnSe:Al/ZnS/ZnS/ZnS QDs, together with a photograph of the QDs under 365 nm UV illumination (inset). (**c**) Time-resolved PL decay curves of different ZnSe quantum dots.

Figure 4 presents the photoluminescence (PL) spectra, photoluminescence excitation (PLE) spectrum, CIE chromaticity diagram, and PL decay curves of the synthesized QDs. As shown in Figure 4a, compared with pristine ZnSe QDs, the band-edge emission of ZnSe:Al QDs at 407 nm is significantly enhanced, accompanied by suppressed defect-related emission centered at ~450 nm, indicating reduced defect-assisted recombination. With sequential growth of ZnS shells, the PL intensity of ZnSe:Al QDs further increased by approximately 80%. Meanwhile, ZnSe/ZnS/ZnS/ZnS QDs also exhibit enhanced PL intensity compared with pristine ZnSe QDs, confirming the passivation effect of the multilayer ZnS shell. The PLE spectrum monitored at 407 nm shows the highest PL response at an excitation wavelength of approximately 375 nm, indicating that excitation at this wavelength most effectively generates the band-edge emission. As shown in Figure 4b, the ZnSe:Al/ZnS/ZnS/ZnS QD powder exhibits bright blue luminescence under 365 nm UV illumination. The corresponding CIE chromaticity coordinates are (0.1557, 0.1335), demonstrating excellent blue color purity.

To further investigate the optical properties of the QDs, PL decay and PLQY measurements were performed. The PL decay curves (Figure 4c) can be well-fitted using a tri-exponential model according to Formula (2). In this equation, A_i_ represents the fitted amplitude of each decay component, while τ_i_ represents the corresponding lifetime component. The relative contribution (α_i_) of each decay component was calculated from the fitted amplitude according to α_i_ = A_i_/(A1 + A_2_ + A_3_). The average lifetime (τ_avg_) was calculated using Formula (3). The detailed fitting parameters are summarized in Table 3. The three decay components represent different carrier relaxation and recombination processes. The fast component (τ_1_) is generally associated with rapid carrier trapping and defect-related nonradiative recombination, while the intermediate component (τ_2_) is mainly related to band-edge excitonic recombination. The long-lived component (τ_3_) is commonly attributed to delayed recombination involving trapped carriers [37]. As summarized in Table 4, a progressive increase in PLQYs from 4.85% to 23.24% and a concomitant extension of τ_avg_ from 52.21 ns to 129.28 ns are observed upon Al doping and subsequent ZnS shell growth, consistent with the enhanced PL intensity.

(2) $$I=A_{1}\exp(-\frac{t}{\tau_{1}})+A_{2}\exp(-\frac{t}{\tau_{2}})+A_{3}\exp(-\frac{t}{\tau_{3}})$$

(3) $$\tau_{avg}=\frac{A_{1}\tau_{1}^{2}+A_{2}\tau_{2}^{2}+A_{3}\tau_{3}^{2}}{A_{1}\tau_{1}+A_{2}\tau_{2}+A_{3}\tau_{3}}$$

The enhanced PL performance of the ZnSe:Al/ZnS/ZnS/ZnS QDs originates from the complementary contributions of Al^3+^ doping and multilayer ZnS shell passivation. Al^3+^ doping primarily regulates core-related defects, while ZnS shells mainly improve surface/interface passivation.

As summarized in Table 4, pristine ZnSe QDs exhibit a relatively low PLQYs of 4.85%, a short average lifetime (τ_avg_) of 52.21 ns, and a low I_band_/I_defect_ ratio of 1.21. In addition, a pronounced defect-related emission centered at approximately 450 nm is observed in the PL spectrum (Figure 4a). These results indicate that a substantial fraction of photogenerated carriers is trapped by defect states and undergoes nonradiative recombination.

**Figure 5:**
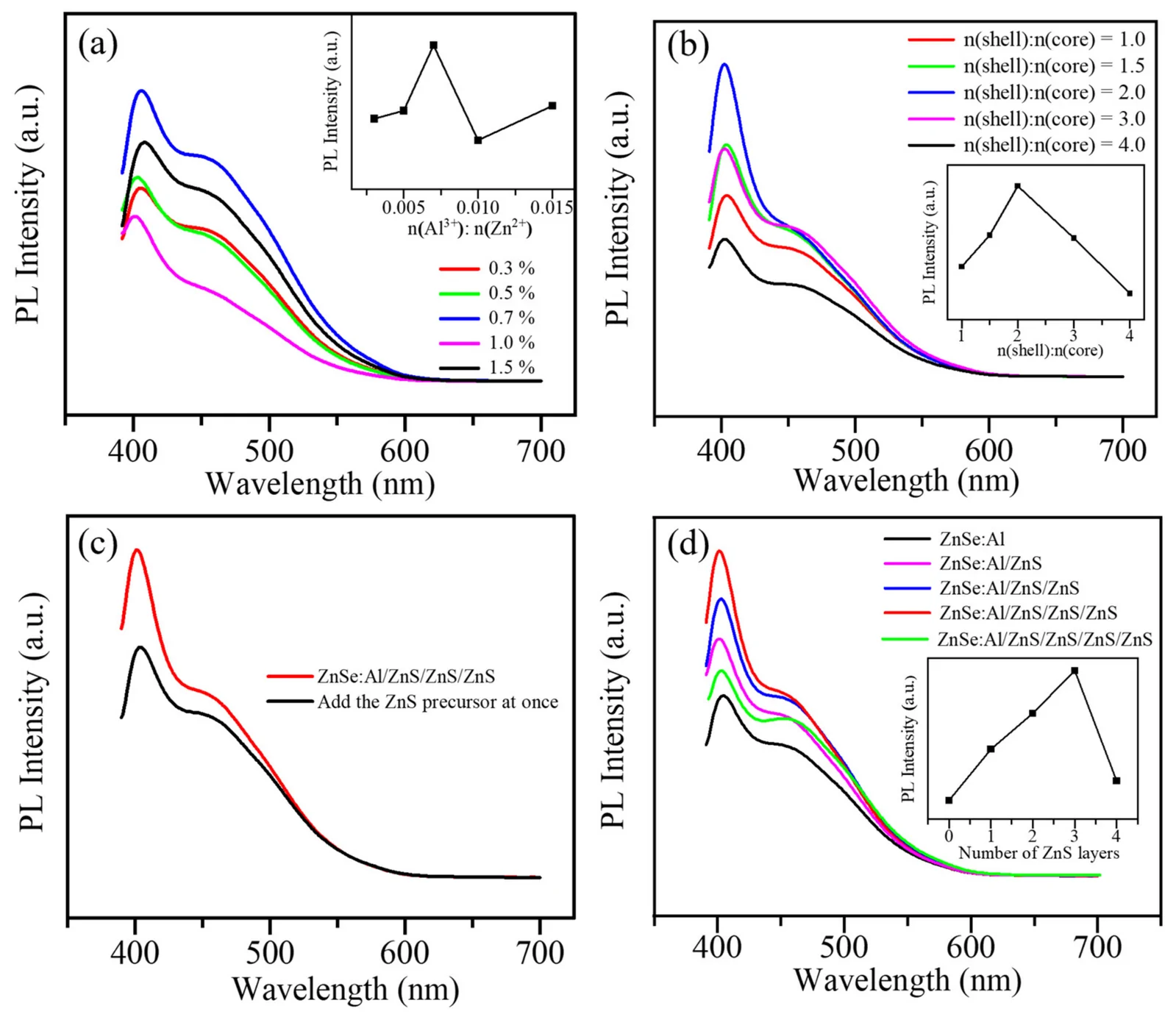
Effects of the synthesis parameters on the photoluminescence properties of ZnSe:Al/ZnS QDs, λ_ex_ = 375 nm. (**a**) Al^3+^ doping concentration. (**b**) Amount of ZnS precursor. (**c**) ZnS precursor addition method. (**d**) Number of ZnS shell layers.

After Al^3+^ doping, the PLQY increased to 12.76%, the τ_avg_ was prolonged to 83.40 ns, and the I_band_/I_defect_ ratio increased to 1.39, indicating a gradual shift in carrier recombination from defect-mediated pathways toward band-edge radiative processes. This evolution is attributed to the regulation of intrinsic defect states by Al^3+^ incorporation, where Al^3+^ incorporation may compensate defect-related states and reduce internal nonradiative recombination. This interpretation is further supported by TRPL analysis, where Al^3+^ incorporation increases the τ_2_ and τ_3_ components while decreasing the contribution of the fast decay component α_1_, indicating reduced fast carrier loss and suppressed defect-mediated nonradiative recombination. As shown in Figure 5a, the PL intensity increases with increasing *n*(Al^3+^)/*n*(Zn^2+^) precursor feeding ratio and reaches the maximum at 0.7%. However, further increasing the Al^3+^ precursor feeding ratio beyond the optimized condition may disturb the optimized defect balance or introduce additional recombination centers, resulting in decreased PL intensity, consistent with the defect-mediated PL optimization behavior commonly observed in doped semiconductor QDs [38,39].

In addition to core defect regulation, ZnS shell growth further modulates exciton recombination by passivating surface trap states and confining carriers. As shown in Figure 5b, the PL intensity increases progressively with increasing ZnS precursor amount, indicating gradual passivation of surface trap states. However, excessive precursor addition leads to a decline in PL intensity, likely due to nonuniform shell growth and the formation of interface defects during shell deposition [40]. This result indicates that shell quality, rather than shell thickness alone, plays a decisive role in PL enhancement.

To overcome the limitations of conventional one-step shell deposition, a stepwise precursor injection strategy was developed in this work. As shown in Figure 5c, the stepwise growth method results in substantially higher PL intensity than the one-step method, since the sequential low-concentration precursor injections facilitate controlled and uniform ZnS shell growth, leading to improved core/shell interface quality and more effective surface defect passivation.

Benefiting from the stepwise injection strategy, the PL performance of ZnSe:Al QDs is progressively enhanced with increasing ZnS shell layers through the gradual suppression of nonradiative recombination pathways. The first ZnS shell delivers the most pronounced improvement, increasing the PLQY from 12.76% to 17.74% and extending the average lifetime from 83.40 to 121.69 ns. The decreased α_1_, together with prolonged τ_2_ and τ_3_ and increased α_2_ and α_3_, indicates effective surface trap passivation and suppressed nonradiative recombination. Upon further growth of the second ZnS shell, the PLQY is further increased to 20.29%, accompanied by a continuous decrease in α_1_, suggesting the gradual elimination of residual carrier trapping pathways. With the growth of the third ZnS shell, the QDs achieve the highest PL performance with a maximum PLQY of 23.24%. The increased I_band_/I_defect_ ratio from 1.55 to 1.82, together with the slight PL blue shift, indicates suppressed defect-assisted recombination and improved carrier confinement [41,42,43]. However, excessive ZnS shell growth introduces additional interfacial defects and deteriorates PL performance (Figure 5d) [44,45].

Consistent with the passivation effect of ZnS shells, ZnSe/ZnS/ZnS/ZnS QDs without Al doping also exhibit improved PL properties compared with pristine ZnSe QDs. More importantly, despite identical ZnS shell structures, ZnSe:Al/ZnS/ZnS/ZnS QDs still exhibit higher PLQY (23.24% vs. 20.83%), longer average lifetime (129.28 vs. 124.56 ns), and an increased I_band_/I_defect_ ratio (1.82 vs. 1.59), indicating that Al^3+^ incorporation provides additional regulation of core-related defect states beyond ZnS shell passivation.

**Figure 6:**
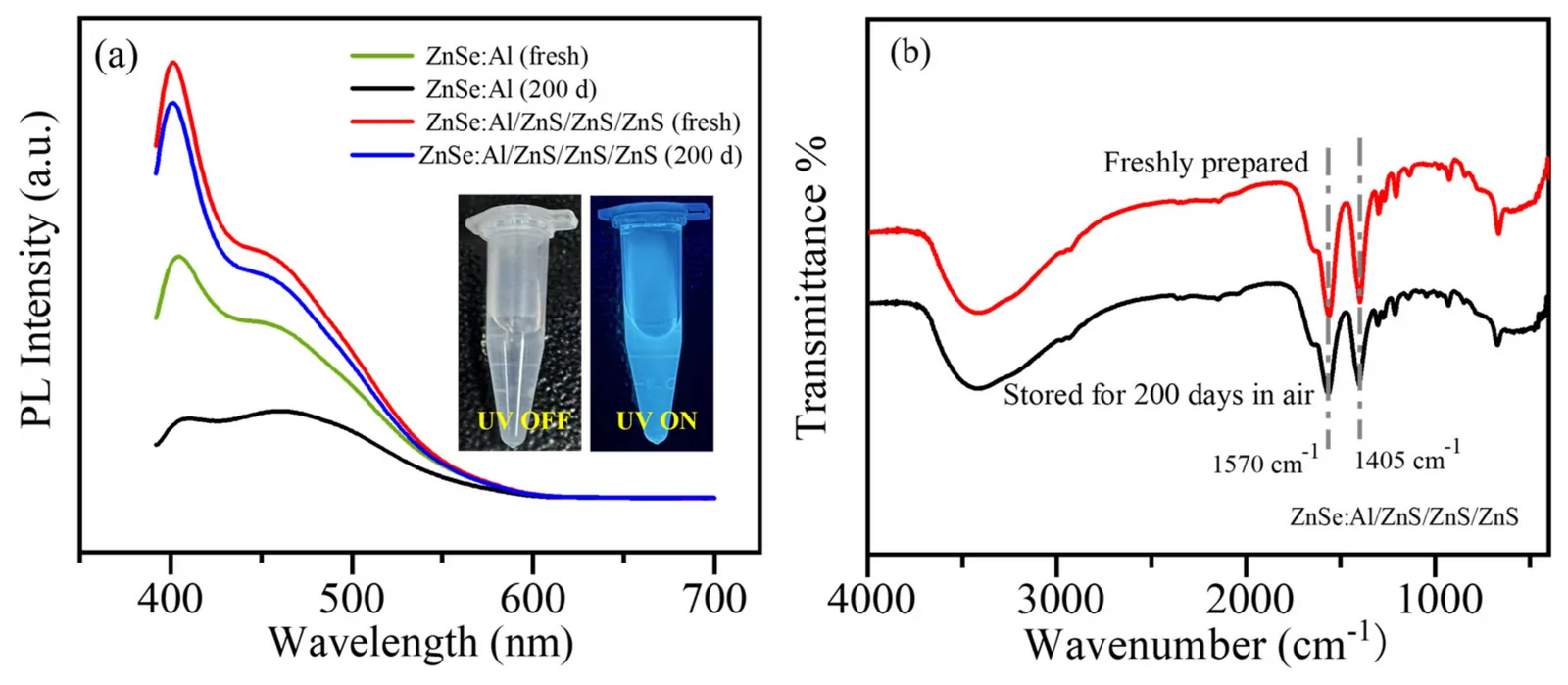
Long-term stability characterization of ZnSe:Al/ZnS/ZnS/ZnS QDs. (**a**) PL spectra of ZnSe:Al and ZnSe:Al/ZnS/ZnS/ZnS QDs after 200 days of air storage (λ_ex_ = 375 nm). The inset shows a photograph of the ZnSe:Al/ZnS/ZnS/ZnS QDs aqueous dispersion under 365 nm UV illumination after 200 days of air storage. (**b**) FT-IR spectra of the ZnSe:Al/ZnS/ZnS/ZnS QDs after 200 days of air storage.

The long-term stability of the prepared QDs were evaluated by monitoring their PL properties after 200 days of storage under ambient conditions (~25 °C, laboratory atmosphere without additional humidity or temperature control). As shown in Figure 6, ZnSe:Al/ZnS/ZnS/ZnS QDs retain approximately 91% of their initial PL intensity, with the I_band_/I_defect_ ratio remaining nearly constant. In contrast, ZnSe:Al QDs retain only 34% of their original PL intensity, accompanied by a decrease in the I_band_/I_defect_ ratio from 1.39 to 0.92. These results indicate that the multilayer ZnS shells effectively protect the ZnSe:Al core from environmental degradation, thereby suppressing the formation of additional defect states and ensuring excellent long-term optical stability.

In addition to optical stability, ZnSe:Al/ZnS/ZnS/ZnS QDs exhibit excellent colloidal stability in aqueous solution. As shown in the inset of Figure 6a, the dispersion remains transparent and continues to emit bright blue luminescence under 365 nm UV excitation even after 200 days, indicating minimal aggregation and preserved emissive properties.

The enhanced colloidal stability is further supported by zeta potential measurements (Table 5). The zeta potential becomes more negative, changing from −33 mV for ZnSe:Al QDs to −46 mV for ZnSe:Al/ZnS/ZnS/ZnS QDs, indicating stronger electrostatic repulsion between particles and improved dispersion in aqueous media [46]. The increased negative zeta potential after sequential ZnS shell growth may result from the modified outer surface chemistry after ZnS formation and changes in the surface-bound MPA ligand environment. These factors may influence the coordination and exposure of negatively charged carboxylate groups (COO^−^), thereby enhancing electrostatic stabilization. The characteristic asymmetric and symmetric stretching vibrations of surface-bound COO^−^ groups at 1570 and 1405 cm^−1^ remain clearly observable after 200 days of air storage, confirming the persistent coordination of MPA ligands on the QD surface (Figure 6b) [47]. The surface-bound carboxyl groups effectively maintain colloidal stability and suppress particle aggregation over extended periods.

## 4. Conclusions

In summary, highly stable water-soluble ZnSe:Al/ZnS/ZnS/ZnS QDs were successfully developed through Al^3+^ doping and a stepwise precursor injection strategy for multilayer ZnS shell growth. The optimized QDs exhibit a PLQYs of 23.24% and an average lifetime (τ_avg_) of 129.28 ns, and retain 91% of their initial PL intensity after 200 days under ambient conditions. The enhanced photoluminescence and stability originate from the synergistic effects of Al^3+^-induced defect regulation and multilayer ZnS shell passivation, which effectively suppress nonradiative recombination and improve carrier confinement. This work provides a feasible strategy for simultaneously regulating core defects and surface states in water-soluble quantum dots, offering a potential approach for designing stable water-soluble semiconductor QDs.

## Figures and Tables

**Table 1:** Reagents used in this work.

Reagents	Purity	Suppliers
Zn(NO_3_)_2_·6H_2_O	≥99%	Chengdu Kelong Chemical Co., Ltd. (Chengdu, China)
Selenium powder (Se)	≥99%	Tianjin Damao Chemical Reagent Factory (Tianjin, China)
NaOH	≥99%	Chengdu Kelong Chemical Co., Ltd.
Zn(OAc)_2_·2H_2_O	≥99%	Tianjin Damao Chemical Reagent Factory
NaBH_4_	≥99%	Tianjin Damao Chemical Reagent Factory
CH_4_N_2_S	≥99%	Tianjin Tianli Chemical Reagent Co., Ltd. (Tianjin, China)
Mercaptopropionic acid	≥99%	Beijing Bailingwei Technology Co., Ltd. (Beijing, China)
Al(NO_3_)_3_·6H_2_O	≥99%	Yinfeng Chemical Reagent Co., Ltd. (Zhengzhou, China)

**Table 2:** Crystallite sizes and lattice parameters of ZnSe and ZnSe:Al QDs estimated from XRD peak fitting.

QDs	FWHM of (111) Peak (°)	Crystallite Size (nm)	Lattice Parameter (Å)
ZnSe	3.767	2.17 ± 0.18	5.668 ± 0.005
ZnSe:Al	5.082	1.61 ± 0.15	5.660 ± 0.006

**Table 3:** Tri-exponential fitting parameters of PL decay curves for ZnSe-based QDs.

QDs	τ_1_ (ns)	α_1_	τ_2_ (ns)	α_2_	τ_3_ (ns)	α_3_	τ_avg_ (ns)
ZnSe	0.88	0.970	17.20	0.023	122.01	0.007	52.21
ZnSe:Al	0.97	0.942	26.17	0.047	161.90	0.011	83.40
ZnSe:Al/ZnS	1.05	0.906	33.04	0.075	206.95	0.019	121.69
ZnSe:Al/ZnS/ZnS	1.36	0.887	34.28	0.089	209.11	0.024	123.74
ZnSe/ZnS/ZnS/ZnS	1.23	0.895	35.05	0.083	209.73	0.022	124.56
ZnSe:Al/ZnS/ZnS/ZnS	1.52	0.865	36.09	0.106	213.44	0.029	129.28

**Table 4:** Photoluminescent quantum yields and I_band_/I_defect_ of prepared ZnSe-based QDs.

QDs	ZnSe	ZnSe:Al	ZnSe:Al/ZnS	ZnSe:Al/ZnS/ZnS	ZnSe:Al/ZnS/ZnS/ZnS
PLQYs	4.85%	12.76%	17.74%	20.29%	23.24%
I_band_/I_defect_	1.21	1.39	1.47	1.55	1.82

**Table 5:** Zeta potential of prepared QDs.

QDs	ZnSe:Al	ZnSe:Al/ZnS	ZnSe:Al/ZnS/ZnS	ZnSe:Al/ZnS/ZnS/ZnS
Zeta potential (mV)	−33	−35.8	−44	−46

## Data Availability

The data that support the findings of this study are openly available in references.
